# Functional study of a KCNH2 mutant: Novel insights on the pathogenesis of the LQT2 syndrome

**DOI:** 10.1111/jcmm.14521

**Published:** 2019-07-30

**Authors:** Roberta De Zio, Andrea Gerbino, Cinzia Forleo, Martino Pepe, Serena Milano, Stefano Favale, Giuseppe Procino, Maria Svelto, Monica Carmosino

**Affiliations:** ^1^ Department of Biosciences, Biotechnologies and Biopharmaceutics University of Bari Bari Italy; ^2^ Cardiology Unit Department of Emergency and Organ Transplantation University of Bari Bari Italy; ^3^ Department of Sciences University of Basilicata Potenza Italy

**Keywords:** arrhythmia, cardiac pathophysiology, channelopathy, electrophysiology, long QT syndrome

## Abstract

The K^+^ voltage‐gated channel subfamily H member 2 (KCNH2) transports the rapid component of the cardiac delayed rectifying K^+^ current. The aim of this study was to characterize the biophysical properties of a C‐terminus‐truncated KCNH2 channel, G1006fs/49 causing long QT syndrome type II in heterozygous members of an Italian family. Mutant carriers underwent clinical workup, including 12‐lead electrocardiogram, transthoracic echocardiography and 24‐hour ECG recording. Electrophysiological experiments compared the biophysical properties of G1006fs/49 with those of KCNH2 both expressed either as homotetramers or as heterotetramers in HEK293 cells. Major findings of this work are as follows: (a) G1006fs/49 is functional at the plasma membrane even when co‐expressed with KCNH2, (b) G1006fs/49 exerts a dominant‐negative effect on KCNH2 conferring specific biophysical properties to the heterotetrameric channel such as a significant delay in the voltage‐sensitive transition to the open state, faster kinetics of both inactivation and recovery from the inactivation and (c) the activation kinetics of the G1006fs/49 heterotetrameric channels is partially restored by a specific KCNH2 activator. The functional characterization of G1006fs/49 homo/heterotetramers provided crucial findings about the pathogenesis of LQTS type II in the mutant carriers, thus providing a new and potential pharmacological strategy.

## 1 INTRODUCTION

The *KCNH2* or *human ether‐a‐go‐go‐related gene 1* (*hERG1*) encodes the Kv11.1 channel α subunit, which underlies the rapidly activating delayed rectifier K^+^ current (IKr) in the heart during phases 2 and 3 of the cardiac action potential, thus playing an important role in cardiac repolarization. Mutations in *KCNH2* induce long QT syndrome type 2 (LQT2), which can lead to ventricular arrhythmias, syncope and sudden death.^1^ The KCNH2 channel contains six transmembrane segments (S1‐S6) with S1‐S4 contributing to the voltage sensor domain (VSD) and S5‐S6 along with the intervening pore loop contributing to the pore domain. The functional channel is a tetramer with the pore domain from each of the four subunits lining the central ion conduction pathway. In addition, the KCNH2 protein contains large cytoplasmic NH2‐terminal and COOH‐terminal domains with several regulatory sites (for review, see 2).

KCNH2 channels can exist in closed, open or inactivated states, and like other voltage‐gated K^+^ channels, they contain multiple positive charges in the S4 domain and this acts as the primary voltage sensor for channel opening.^3^ The inactivation process of KCNH2 channels, however, exhibits several unusual features: the kinetics of inactivation is much more rapid than the kinetics of activation,^4^ and the inactivation process is voltage‐dependent.^5^ This unique combination of biophysical properties underlies the physiological role that KCNH2 plays in cardiac repolarization.

About 300 mutations in the *KCNH2* gene have been identified to date (http://www.fsm.it/cardmoc/). A variety of mechanisms underlie the dysfunction of the KCNH2 channel in these mutants, including the failure of the mutant channels to reach the cell surface, defective activation/inactivation, loss in permeation selectivity and dominant‐negative suppression of the function of wild‐type channels.^6^ In LQTS2, syncope is often the first presenting symptom and the risk of sudden cardiac death is very high. Genetic testing can give invaluable information to support diagnosis in the patient and prognosis in familiars and to address treatment in both. The management of syncope survivors and relatives at high risk often consists in the use of an implantable cardioverter defibrillator (ICD) to correct or interrupt life‐threatening arrhythmias.

In LQTSs, general emotional stress and intense exercise play a prominent role in triggering ventricular arrhythmias. Thus, the only pharmacological approach so far consists of the use of β‐adrenoceptor antagonists to reduce sympathetic or adrenergic stimulation.^7^ However, as β‐blocker efficacy is variable, ICD is the only treatment proved effective in treating the resulting arrhythmia and preventing sudden death in patients affected by LQTSs. Indeed, the pharmacotherapy in LQTSs is a challenging issue in the actual clinical management and deciphering the exact mutation‐specific pathogenetic mechanism is fundamental to design therapies tailored upon patients’ requirements.

Here, we characterized by electrophysiology the biophysical properties of a C‐terminus‐truncated mutant of KCNH2 (G1006fs/49) that causes QT interval prolongation in members of an Italian family. We identify new insights regarding the role of the C‐terminal tail of the KCNH2 channel, the pathogenetic mechanisms underlying the LQTS type II identified in the mutant carriers and a possible pharmacological strategy.

## 2 MATERIALS AND METHODS

### 2.1 Patients

The heterozygous c.3017delG mutation in *KCNH2* gene (NM_000238) exon 13, identified for the first time in 2003 (www.ncbi.nlm.nih.gov/variation/tools/1000genomes/?gts=rs794728504),^8^ has been found in five members of an Italian family screened in our Clinical Unit dedicated to cardiomyopathies. The mutation consists of the G deletion at position 3017 in the *KCNH2* coding region, causing both a translational frame shift starting from the glycine 1006 and a premature protein termination 49 amino acids forward. This KCNH2 variant will be indicated throughout the manuscript as G1006fs/49.

All participants underwent clinical workup, including medical history, physical examination, 12‐lead electrocardiogram (ECG), transthoracic echocardiography and 24‐hour ECG recording. Written informed consent was obtained from all patients or parents of minors included in the study. This project conforms to the principles outlined in the Declaration of Helsinki and was approved by the Ethics Committee of University Hospital Consortium, Policlinico of Bari, Italy.

### 2.2 KCNH2 channel expression in HEK293 cells

HEK293 (human embryonic kidney, ATCC® CRL‐1573™) cells were grown in Dulbecco's modified Eagle medium (DMEM) GlutaMAX® (Thermo Fisher Scientific) supplemented with 10% of FBS (Thermo Fisher Scientific) and 1% of penicillin‐streptomycin (Thermo Fisher Scientific, Waltham) at 37°C with 5% di CO_2_.

For the expression of both wild‐type and mutant KCNH2 channels in HEK293 cell, two constructs for protein expression in mammalian systems provided by VectorBuilder (Cyagen Biosciences) were used. The cDNA of the human wild‐type channel was expressed in frame at the N‐terminal tail with the Em‐GFP tag protein (KCNH2; Vector ID: VB161014‐1085qzg). The cDNA of the c.3017delG variant in *KCNH2* gene was expressed in frame at the N‐terminal tail with the mCherry tag protein (G1006fs/49; Vector ID: VB161014‐1086gyz).

For both confocal microscopy, Western blotting and electrophysiological experiments, HEK293 cells plated in one well of a 6‐multiwell cell culture support at 70%‐90% of confluence were transiently transfected with KCNH2 and/or the mutant G1006fs/49 constructs using Lipofectamine 2000 (Invitrogen Corporation). Four microgram of DNA and 10 μL of lipofectamine in 250 μL of OPTIMEM (Gibco™ Opti‐MEM™) medium were used for each well as reported on the official guide of Lipofectamine 2000 (Invitrogen Corporation). The efficiency of transfection was estimated of about 60%.

### 2.3 Confocal analysis

Transfected cells were plated on sterile 15‐mm‐diameter coverslips (Thermo Fisher Scientific) coated before cell plating with Poly‐L‐Lys for 20 minutes at RT (Sigma‐Aldrich). After adhesion, the cells were fixed in ice‐cold 100% methanol (Sigma‐Aldrich) for 5 minutes. The coverslip was mounted upside down on a microscope slide using a drop of mounting medium composed by 50% PBS 2×, 50% glycerol 100% and 1% m/v n‐propyl gallate; pH = 8. Confocal images were obtained with a confocal laser‐scanning fluorescence microscope (Leica TSC‐SP2).

### 2.4 Western blot analysis

Cell lysates were obtained as follows: transiently transfected HEK293 cells were placed on ice, washed in PBS and exposed to RIPA buffer (NaCl 150 mmol/L, Tris/HCl 10 mmol/L, 1% Triton X‐100, SDS 0.1%, deoxycholate‐Na 1%, EDTA 5 mmol/L; pH 7.2) supplemented with protease and phosphatase inhibitors (NaF 10 mmol/L, orthovanadate 100 mmol/L, pyrophosphate 15 mmol/L). Cells were then sonicated at 60 Amplitude with Vibra‐cell® (Sonics & Materials Inc) and membranes pelleted at 13 000 rpm for 30 minutes at 4°C. Ten microlitre of supernatant was denatured in 1× Laemmli Sample Buffer (Bio‐Rad), 50 mmol/L DTT and electrophoresed on 7.5% polyacrylamide SDS gel (TGX Stain‐Free™ FastCast™ Acrylamide Kit, 7.5%; Bio‐Rad). After transfer on Immobilon ®‐P, the membrane was subjected to Western blotting in TBST‐BSA 3% (TBST: 50 mmol/L Tris, 150 mmol/L NaCl, 0.1% Tween‐20; pH 7.4) or TBST‐5% milk for 2 hours, depending on primary antibodies used.

KCNH2 channel was identified using a mouse anti‐GFP monoclonal antibody (1:1000 in TBST‐5% milk, GF28R; Thermo Fisher Scientific) while, G1006fs/49 channel, we used a rabbit anti‐mCherry polyclonal antibody (1:1000 in TBST‐BSA 1%, ab167453; Abcam). The following secondary antibodies were used: the goat antimouse IgG‐HPR conjugate (1:5000 in TBST‐5% milk; Bio‐Rad) for membranes previously incubated with anti‐GFP and an anti‐rabbit IgG‐peroxidase antibody produced in goat (1:5000 in TBST‐BSA 3%; Sigma‐Aldrich) for membranes previously incubated with anti‐mCherry. The immunoreactive bands were detected with a ChemiDoc™ System (Bio‐Rad). Densitometric analysis was performed by Image Lab 6.0 software. For protein load controls, the stain‐free technology from Bio‐Rad was used.

### 2.5 Electrophysiological recordings

Transiently transfected HEK293 cells were trypsinized and plated on 35‐mm petri dishes coated with Poly‐L‐Lys (20 minutes at room temperature; Sigma‐Aldrich) 3‐4 hours before the experiments at about 30% of confluence.

All recordings were performed at room temperature in whole‐cell configuration and voltage clamp mode using a system integrated by Crisel Instruments (Crisel Instruments) including a MultiClamp 700B amplifier (Axon Instrument‐Molecular Devices) connected to an Axon Digidata 1500 (Axon Instrument‐Molecular Devices). Currents were sampled at 10 kHz and low‐pass filtered at 5 kHz. After gigaseal formation and whole‐cell access, pipette capacitance (Cp) and the membrane capacitance (Cm) were compensated adjusting the Cp Fast and the Cp Slow setting on the MultiClamp 700B. Series resistance (Rs) was monitored during the experiments. A Rs stable for the entire experiment and lower than 20 MΩ was considered acceptable.

The experiments were performed using borosilicate pipettes pulled with a P‐1000 puller (Sutter Instrument, Novato) with resistance of 3‐5 MΩ when filled with an internal pipette solution contained (in mmol/L): 100K‐aspartate, 20 KCl, 2 MgCl_2_, 10 EGTA, 10 HEPES, 1 CaCl_2_, pH 7.2 with KOH. The extracellular solution contained (in mmol/L): 140 NaCl, 10 KCl, 1 MgCl_2_, 1 CaCl_2_, 5 glucose, 10 HEPES, pH 7.4 with NaOH. We used an extracellular solution with higher K^+^ concentration (10 mmol/L) to evoke a more enhanced tail current.

The effect of perfusion with 30 µmol/L NS1643 on the KCNH2 current was investigated after recording the current in normal extracellular solution.

AxoScope 10.4 (Molecular Devices) and pClamp 10.4 (Molecular Devices) were used to acquire and analyse the data.

### 2.6 Statistical analysis

GraphPad Prism 6 was used for the statistical analysis and graph representation of the electrophysiological data. Data are given as mean ± standard error of the mean. Statistical analysis was performed using two‐way ANOVA test for multiple comparisons.

## 3 RESULTS

### 3.1 Clinical data of the Italian family carrying the G1006fs/49 variant

The heterozygous c.3017delG mutation in *KCNH2* gene consists of the G deletion at position 3017 in the *KCNH2* coding region, causing both a translational frame shift starting from the glycine 1006 and a premature protein termination 49 amino acids forward; indeed, the KCNH2 variant will be indicated throughout the manuscript as G1006fs/49.

**Figure 1:**
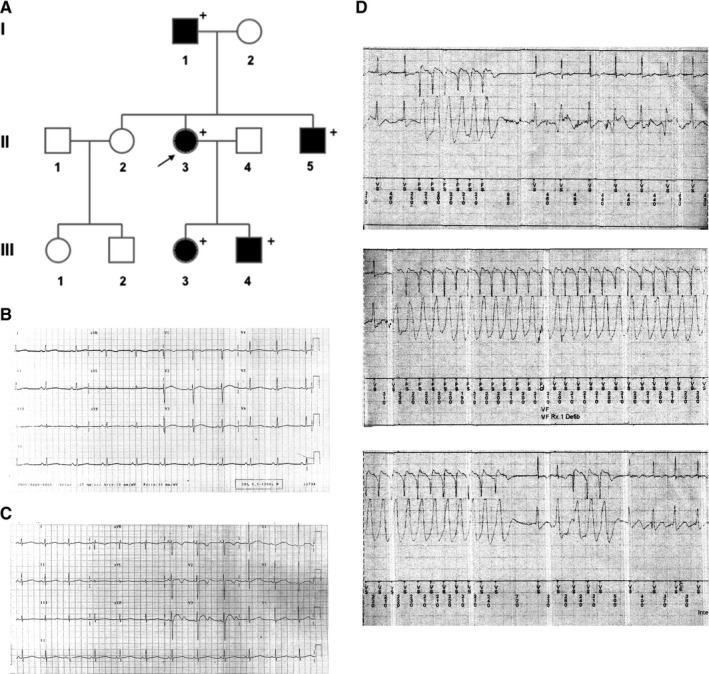
A, Pedigree of the family carrying the G1006fs/49 mutant of the KCNH2 channel. Filled symbols indicate clinically affected individuals; + indicates positive for the mutation; arrow indicates the index patient. B, The index patient and (C) her brother had prolonged QT intervals corrected for heart rate (QTc) on electrocardiogram. D, The index patient was treated with beta‐blocker therapy and received an implantable cardioverter defibrillator (ICD) but continued to exhibit tachyarrhythmic episodes that were recorded on ICD interrogation

As shown in Figure 1A, the pedigree of the family, spanning 3 generations, was consistent with an autosomal dominant transmission. The genetic screening has been performed in five family members and all of them resulted positive for the mutation (+ in the pedigree). The clinical features co‐segregating with the G1006fs/49 variant are collected in the Table S1. The proband (subject II‐3) aged 23 years, after sudden awakening from sleep following a loud noise (her brother's intense crying), suffered from cardiac arrest due to ventricular fibrillation resuscitated by transthoracic DC shock. During the same night, her brother (subject III‐5, aged 11 years) experienced syncope (with fall to the ground associated with trauma to the right cheekbone). ECGs from the proband (Figure 1B) and her brother (Figure 1C) showed prolonged corrected QT intervals (QTc). Both received an implantable cardioverter defibrillator (ICD). All mutant carriers were treated with β‐blocker therapy (nadolol). During a follow‐up of 15 years, the proband suffered from episodes of palpitations related to emotions and, 11 years after ICD implant, she had a syncope triggered by intense emotional stress. Rapid rate non‐sustained ventricular tachycardia episodes were recorded and reported by ICD (Figure 1D).

### 3.2 Confocal microscopy analysis and Western blotting

**Figure 2:**
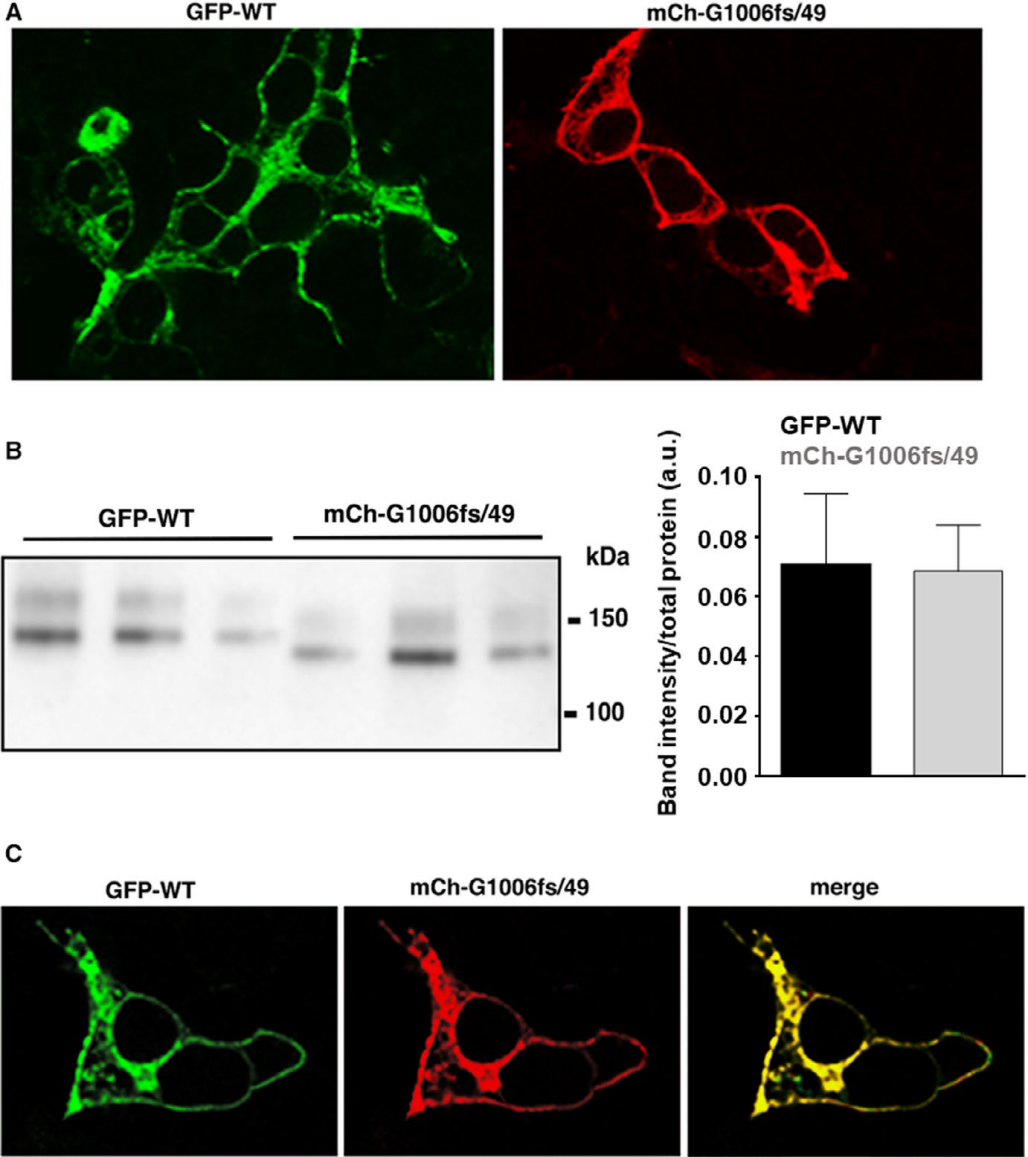
A, Confocal analysis of HEK293 cell expressing either GFP‐tagged KCNH2 homotetramers (KCNH2) or mCherry‐tagged G1006fs/49 homotetramers (G1006fs/49). B, Western blotting analysis using cell lysate of HEK293 cells expressing either GPP‐tagged KCNH2 homotetramers (KCNH2) or mCherry‐tagged G1006fs/49 homotetramers (G1006fs/49). The bands were detected using the antibodies against the fluorescent tags. C, Densitometric analysis performed on 3 independent Western blotting experiments. D, Confocal analysis of HEK293 cells expressing simultaneously GFP‐tagged KCNH2 channels (KCNH2) and mCherry‐tagged G1006fs/49 channels (G1006fs/49)

Confocal images showed that both KCNH2 and G1006fs/49 homotetramers were able to reach the plasma membrane (Figure 2A), although trafficking portions were also visible for both proteins. Western blotting of HEK293 cells expressing either KCNH2 or G1006fs/49 showed that both proteins were correctly processed in the biosynthetic pathway as showed by the presence of immature and mature proteins of about 170 and 190 kD, respectively. The molecular weights of the two bands consider the presence of the fluorescent tags in both proteins. The migration of the bands corresponding to the G1006fs/49 protein resulted delayed compared to those of KCNH2 protein since the absence of about 100 amino acids in the G1006fs/49 (Figure 2B). The expression levels of KCNH2 and G1006fs/49 were comparable in HEK cell lysates (Figure 2C). When expressed simultaneously in HEK293 cells, KCNH2 and G1006fs/49 largely colocalize at the plasma membrane and within the intracellular compartments (Figure 2D, merge).

### 3.3 Electrophysiological analysis

Patch clamp, whole‐cell experiments in voltage clamp mode were performed to compare the biophysical properties of the G1006fs/49 channels with those of either KCNH2 channels expressed either as homotetramers or as heterotetramers in HEK293 cells. Isolated, transiently transfected cells were selected for the recordings according to the expression of GFP for KCNH2 and mCherry for G1006fs/49.

#### 3.3.1 Activation kinetics

**Figure 3:**
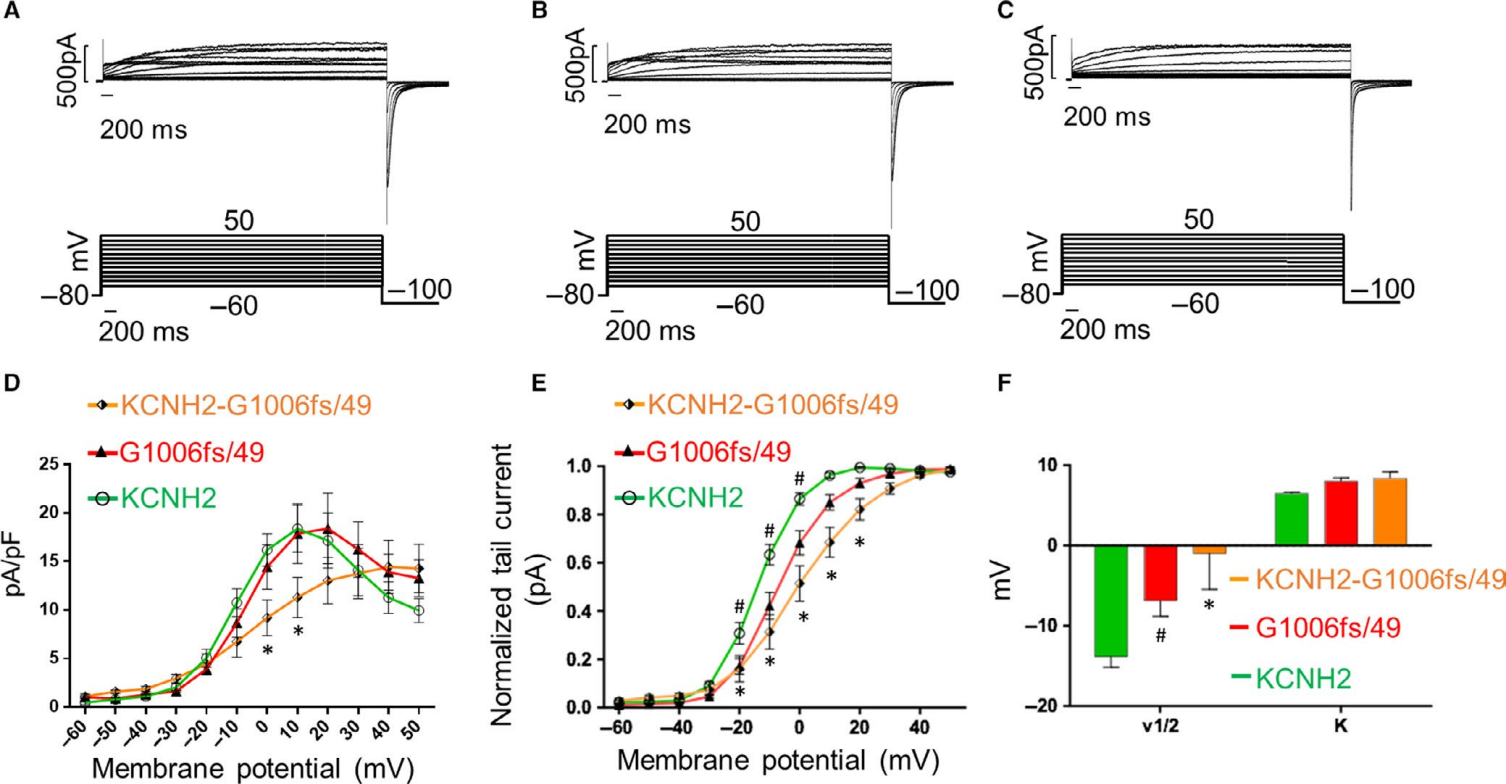
K^+^ currents recorded for (A) KCNH2 homotetramers, (B) G1006fs/49 homotetramers and (C) KCNH2‐G1006fs/49 heterotetramers in HEK293 cells. D, I‐V plot of the K^+^ currents measured at the end of the depolarizing steps for KCNH2 homotetramers (green trace, cells = 10), G1006fs/49 homotetramers (red trace, cells = 9) and KCNH2‐G1006fs/49 heterotetramers (orange trace, cells = 10). E, Activation curves measured with K^+^ normalized tail currents fitted to a Boltzmann relationship. ANOVA test, *P* ≤ 0.05; *KCNH2 vs KCNH2‐G1006fs/49; ^#^KCNH2 vs G1006fs/49. F, Representation of *V*_1/2_ and κ values. ANOVA test, *P* ≤ 0.05; *KCNH2 vs KCNH2‐G1006fs/49; ^#^KCNH2 vs G1006fs/49

KCNH2 current was elicited and recorded using a voltage protocol consisting of a holding potential of −80 mV, 4s depolarizing steps from −60 to +50 mV (with increments of 10 mV), and a third step at −100 mV, used to record the tail current.^4^, ^9^, ^10^ Figure 3 shows the voltage clamp protocol used to investigate the activation kinetics of the K^+^ current and the original current records obtained from KCNH2 homotetramers (A), G1006fs/49 homotetramers (B) and KCNH2‐G1006fs/49 heterotetramers (C). In response to the depolarizing steps, outward currents were evoked with similar amplitudes in all conditions.

Figure 3D shows the *I‐V* plot of the evoked outward K^+^ current measured at the end of the depolarizing steps. The K^+^ current increased at each further depolarizing step until reached similar maximum outward current amplitude at +10 mV in KCNH2 (18.378 ± 2.418 pA/pF) and at +20 mV in G1006fs/49 homotetramers (18.416 ± 3.630 pA/pF), respectively. After reaching the peak, both *I‐V* curves quickly decreased at more positive potentials because of the voltage‐dependent KCNH2 channel inactivation. When KCNH2 and G1006fs/49 channels were co‐expressed in the same cells, the *I‐V* curve reached the maximum value of current amplitude at +40mV. Although the maximum outward current recorded for KCNH2‐G1006fs/49 heterotetramers (14.438 ± 2.740 pA/pF) was reached at more positive potential, the maximal outward current was not statistically different than those recorded for either KCNH2 or G1006fs/49 homotetramers. These results suggest that the G1006fs/49 homotetramers and more strikingly the KCNH2‐G1006fs/49 heterotetramers have a delay in the voltage sensitivity of the activation, although they have a functional transporting pore.

After every depolarizing step, a further hyperpolarization at −100 mV allowed the recording of the tail current and used to construct the activation curve of the KCNH2 currents. The activation curves in Figure 3E were obtained fitting to a Boltzmann relationship of the K^+^ tail current normalized to the maximum tail current amplitude.

$$f\left(V\right)=\frac{I_{\max}}{1+e^{\left(V_{\text{mid}}-V\right)/V_{c}}}$$

The activation curve of KCNH2 homotetramers confirmed a threshold voltage close to −40 mV and that it was fully activated around +10 mV with a half‐maximum activation voltage (*V*_1/2_) of −13.082 ± 1.368 mV and a slope factor (κ) of 6.485 ± 0.182 (Figure 3E,F, green trace). On the other hand, G1006fs/49 homotetramers exhibited a threshold voltage of around −20 mV and were fully activated at near +20 mV with a *V*_1/2_ of −6.851 ± 1.984 mV and a slope factor (κ) of 8.022 ± 0.406 (Figure 3F, red trace). Indeed, the normalized tail current from −20 mV to +0 mV and the half‐maximum activation voltage (*V*_1/2_) were significantly right‐shifted for G1006fs/49 homotetramers compared to the KCNH2 homotetramers (Figure 3E,F, green and red traces). In addition, the current of KCNH2‐G1006fs/49 heterotetramers (orange trace) showed a threshold voltage around −40 mV and a fully activation close to +40 mV, with a *V*_1/2_ of −0.976 ± 4.49 mV a slope factor (κ) of 8.4 ± 0.755 (Figure 3F, orange trace).

Indeed, the normalized tail current from −20 to +20 mV and the half‐maximum activation voltage (*V*_1/2_) resulted significantly right‐shifted towards positive voltages in the KCNH2‐G1006fs/49 heterotetrameric channels compared with KCNH2 homotetramers.

#### 3.3.2 Inactivation kinetics

**Figure 4:**
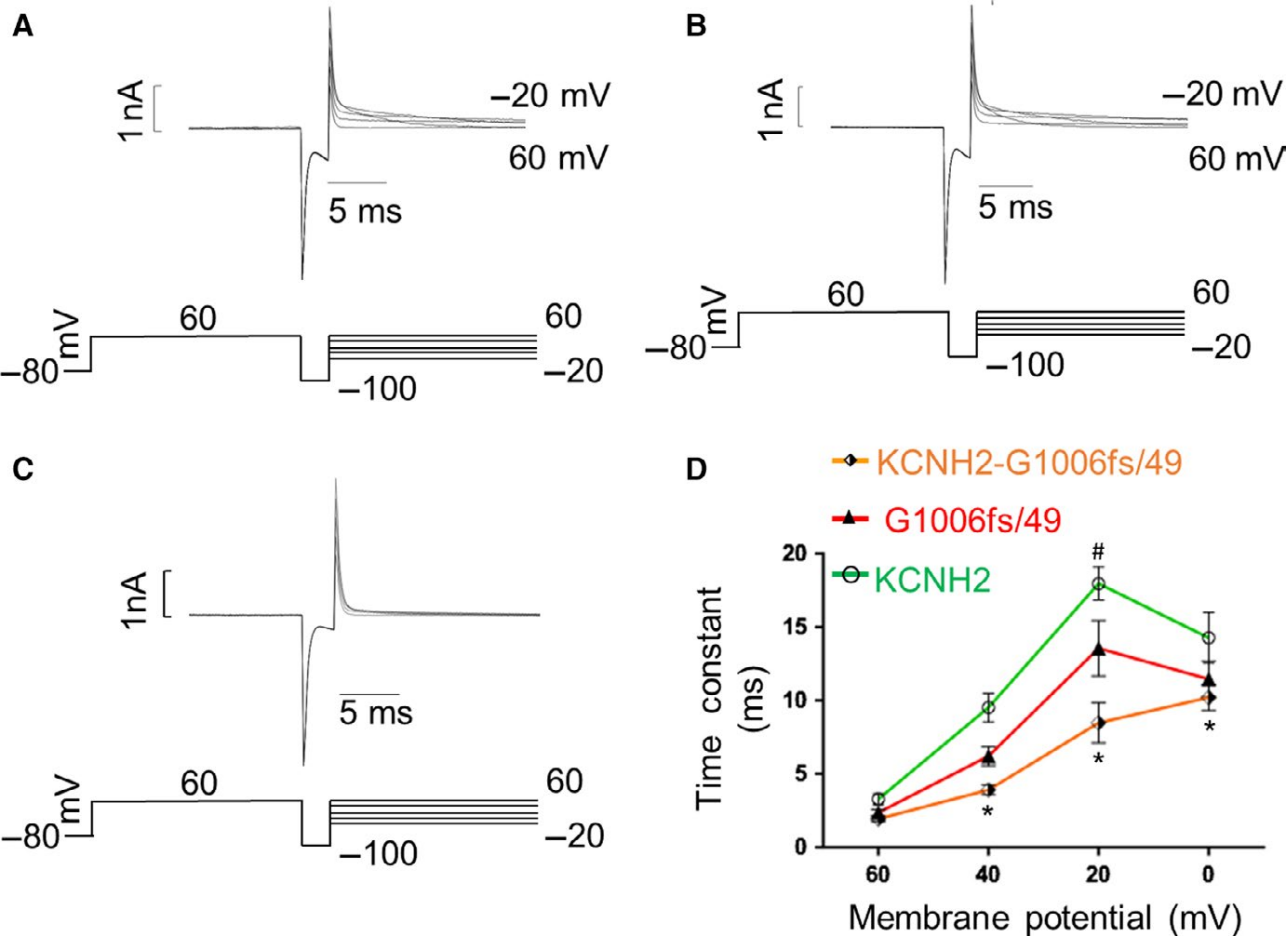
Voltage clamp protocol used to analyse channel inactivation and relative traces recorded for (A) KCNH2 homotetramers, (B) G1006fs/49 homotetramers and (C) KCNH2‐G1006fs/49 heterotetramers in HEK293 cells. D, Representation of the time constant of inactivation (tau) vs membrane potential obtained by fitting the current with a single‐exponential function for KCNH2 homotetramers (green trace, cells = 4), G1006fs/49 homotetramers (red trace, cells = 4) and KCNH2‐G1006fs/49 heterotetramers (orange trace, cells = 4). ANOVA test, *P* ≤ 0.05; *KCNH2 vs KCNH2‐G1006fs/49; ^#^KCNH2 vs G1006fs/49

KCNH2 inactivation is well known to proceed faster than channel activation; thus, its investigation required a three‐pulse voltage protocol.^4^, ^9^, ^10^ The currents were activated (and inactivated) by a 200‐ms step to +60 mV. Then, the hyperpolarization of the membrane for 2 ms at −100 mV was used to allow the recovery from inactivation and finally a second set depolarizing steps at voltages between −20 mV and +60 mV allowed the elicitation of an instantaneous current, large in amplitude and rapidly inactivated (Figure 4A,B,C). The time constant of inactivation was obtained by fitting the decay of the current (excluding the capacitance spike) with a single‐exponential function.

$$f\left(t\right)=\sum_{i=1}^{n}A_{i}e^{-t/\tau }+C$$

Figure 4 shows the voltage clamp protocol used to investigate the inactivation kinetics of the KCNH2 currents and the original current records obtained for KCNH2 homotetramers (A), G1006fs/49 homotetramers (B) and KCNH2‐G1006fs/49 heterotetramers (C). Figure 4D shows the average of the time constant of inactivation measured at voltages between 0 and +60 mV for each type of channels. G1006fs/49 channels (red trace) were characterized by a faster inactivation compared to KCNH2 homotetramers at +20 mV, which was drastically enhanced in KCNH2‐G1006fs/49 heterotetramers at all voltages tested.

#### 3.3.3 Recovery from inactivation

As previously reported,^4^, ^9^, ^10^ once activated by depolarization, KCNH2 channels exhibited a small and slow outward current (see also Activation kinetics3.3.1 paragraph). At positive voltages, KCNH2 is turned off by an inactivation mechanism much faster than activation. They can quickly recover from inactivation at negative potentials producing a fast‐inward current (larger than the outward) before return to the closed deactivated state. Considering these KCNH2 biophysical properties, we determined the time constant of inactivation using a two‐step voltage clamp protocol consisting of a holding potential of −80 mV, a first step at +60 mV used to activate and inactivate the channel and a second step at voltages between −100 and −20 mV used to induce a rapid recovery from inactivation. The time constant of recovery from inactivation was obtained using the same single‐exponential function described above for fitting the rising phase of the currents evoked at −20 and −40 mV where the deactivation is too slow to contribute to the kinetics of the current recorded. At more negative potentials (−60 to −100 mV), where the deactivation overlaps with the recovery from inactivation, the time constant was obtained using a double‐exponential function.

**Figure 5:**
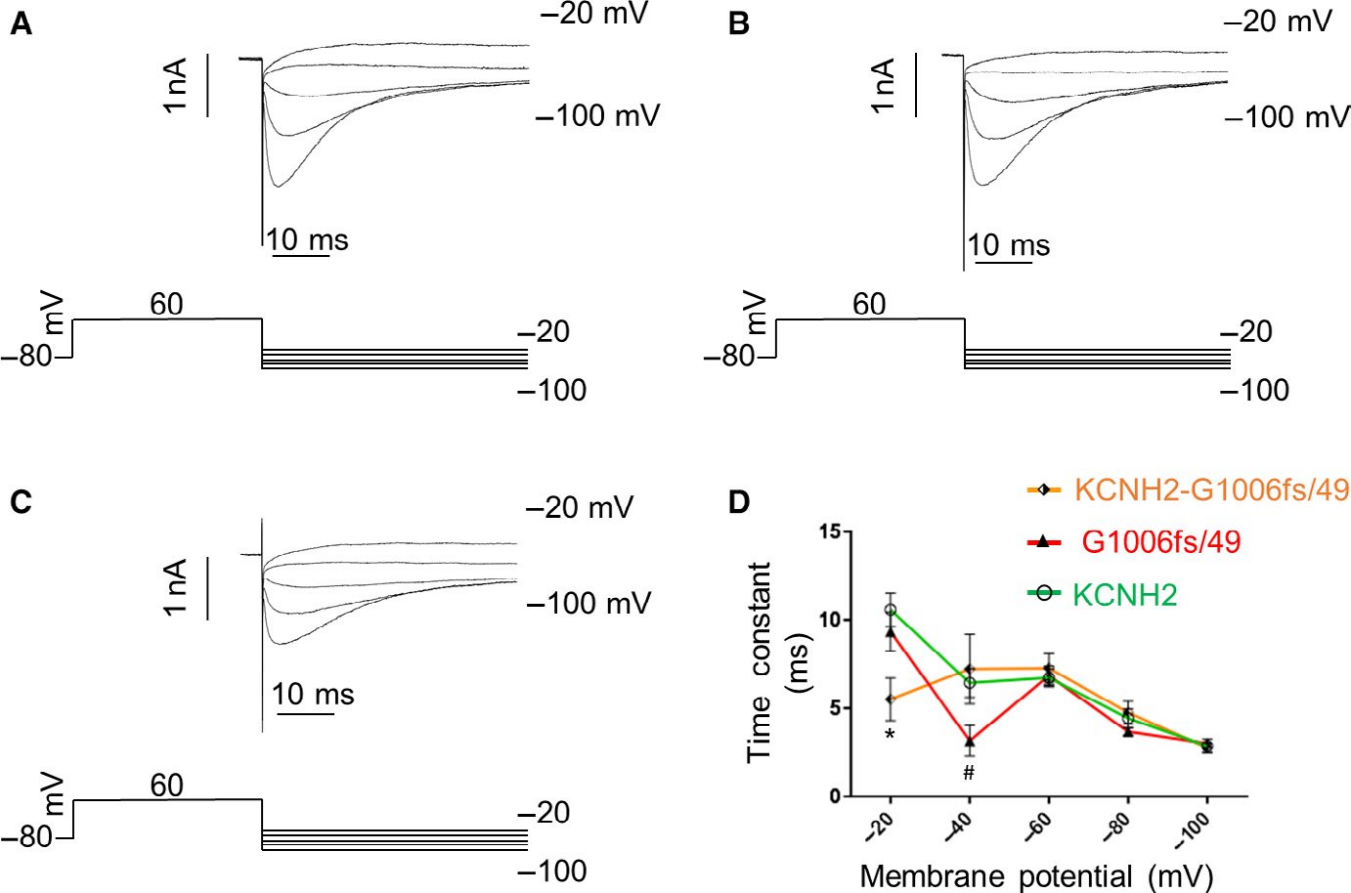
Voltage clamp protocol used to analyse channel recovery from inactivation and 100 ms of a relative trace recorded for (A) KCNH2 homotetramers, (B) G1006fs/49 homotetramers and (C) KCNH2‐G1006fs/49 heterotetramers in HEK293 cells. D, Time constant of inactivation over the membrane potential of KCNH2 homotetramers (green trace, cells = 7), G1006fs/49 homotetramers (red trace, cells = 6) and KCNH2‐G1006fs/49 heterotetramers (orange trace, cells = 8). ANOVA test, *P* ≤ 0.05; ^#^KCNH2 vs G1006fs/49; *KCNH2 vs KCNH2‐G1006fs/49

The recovery from inactivation was investigated for either KCNH2 (Figure 5A), G1006fs/49 homotetramers (Figure 5B) or KCNH2‐G1006fs/49 heterotetramers (Figure 5C) and plotted as mean ± SEM in Figure 5D. We found that for potentials more positive than −60 mV, the recovery from the inactivation was significantly faster in either G1006fs/49 homotetramers (at −40 mV, red trace) or KCNH2‐G1006fs/49 heterotetramers (at −20 mV, orange trace) compared to KCNH2 homotetramers (green trace). Rates of deactivation resulted comparable in all the experimental conditions (data not shown).

#### 3.3.4 Effect of NS1643 on KCNH2 currents

**Figure 6:**
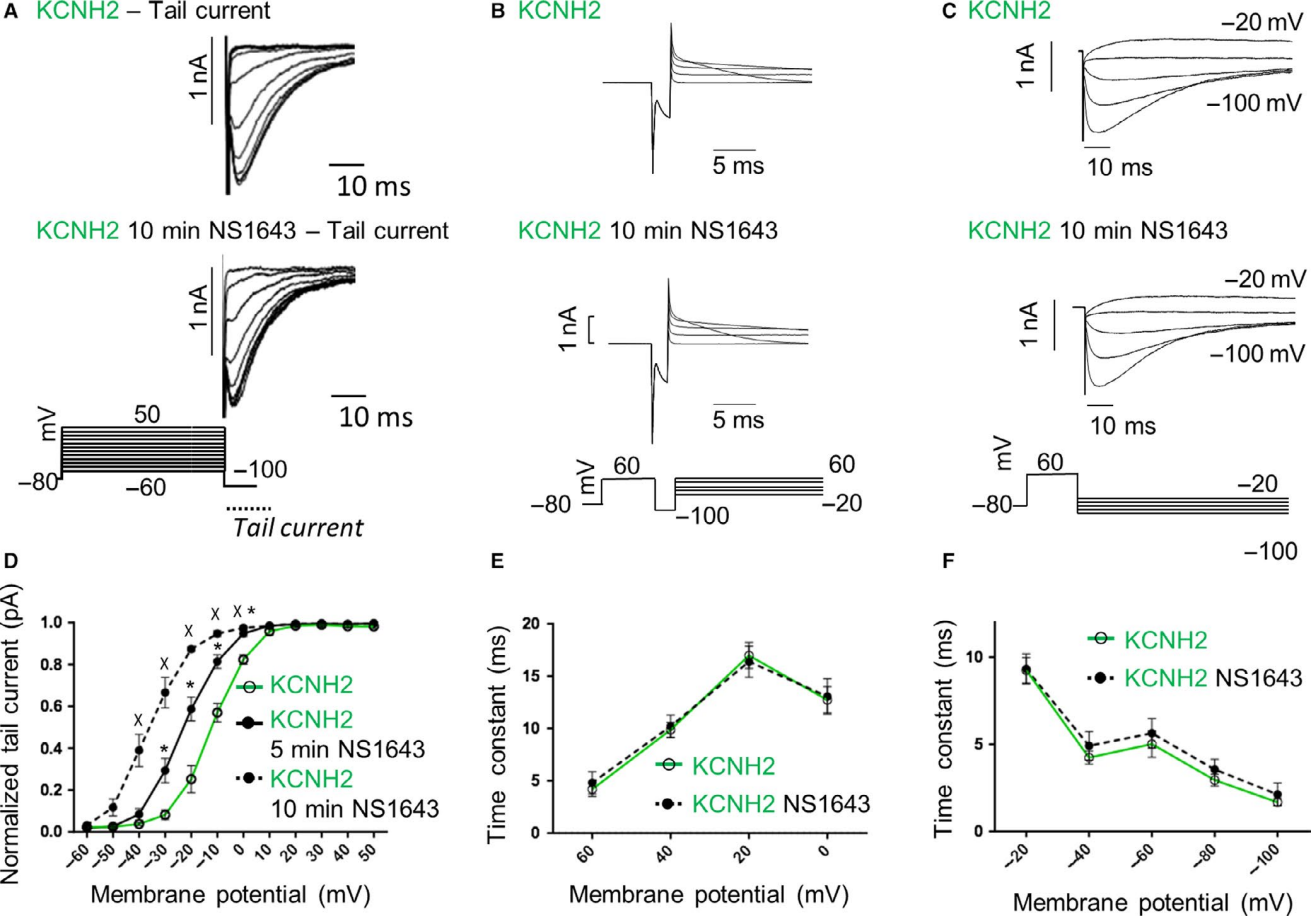
Representative traces of the KCNH2 tail currents with and without NS1643 (A), representative traces of KCNH2 currents used for evaluating the channel inactivation rate with and without NS1643 (B), representative traces of KCNH2 currents used for evaluating the rate of recovery from inactivation with and without NS1643 (C). The activation curve, (D) the time constant of inactivation (E) and recovery from inactivation (F) for KCNH2 homotetramers either in control extracellular solution (green trace, cells = 6) or after administration by perfusion of NS1643 30 µmol/L (black traces, cells = 6). ANOVA test, *P* ≤ 0.05, *KCNH2 vs KCNH2 5 min NS1643, ^X^KCNH2 vs KCNH2 10 min NS1643

First, the effect of the specific agonist NS1643^11^ was investigated on KCNH2 channels activation, inactivation and recovery from inactivation as described in the previous paragraphs (see paragraphs Activation kinetics3.3.1, Inactivation kinetics3.3.2 and Recovery from inactivation3.3.3). Figure 6D shows the activation curves of KCNH2 homotetramers obtained recording the tail current at −100mV in control extracellular solution (green trace) and after 5 minutes (continuous black trace) or 10 minutes (dashed black trace) of continuous exposure to 30 µmol/L NS1643. *V*_1/2_ of activation was of −13.21 ± 1.248 mV in control extracellular solution (green trace), −22.9 ± 1.321 mV after 5‐minute (continuous black trace) and −34.14 ± 2.131 mV after 10‐minute exposure (dashed black trace) to 30 µmol/L NS1643. Under these experimental conditions, KCNH2 channels’ activation curve was significantly left‐shifted of about 10 mV after 5 minutes and 20 mV after 10 minutes of NS1643 treatment, respectively. The slope of the current (κ) was unaffected (control extracellular solution, κ = 6.416 ± 0.163; 5 minutes 30µmol/L NS1643, κ = 6.392 ± 0.2; 10 minutes 30µmol/L NS1643, κ = 6.431 ± 0.175). Interestingly, NS1643 did not modulate the other biophysics properties of the KCNH2 channels such as the inactivation kinetics and recovery from the inactivation (Figure 6E,F). Given the ability of NS1643 to shift towards less positive voltages the activation curve of KCNH2 homotetramers, we analysed its effect on the activation kinetics of both G1006fs/49 homotetramers and KCNH2‐G1006fs/49 heterotetramers.

**Figure 7:**
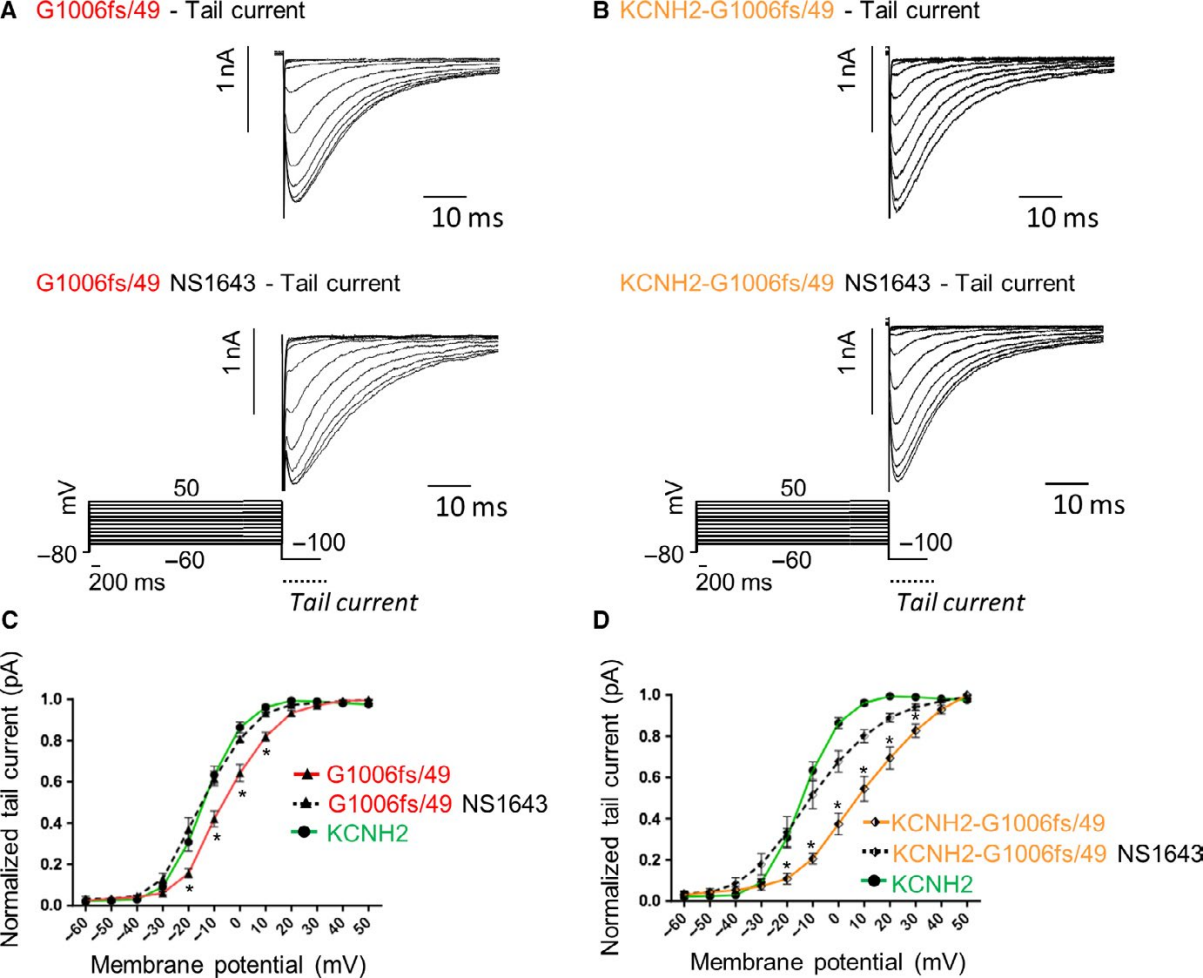
Representative traces of the G1006fs/49 (A) and KCNH2‐G1006fs/49 (B) tail currents with and without NS1643 used for evaluating the channel activation kinetics. Effect of 30 µmol/L NS1643 on (C) G1006fs/49 homotetramers and (D) KCNH2‐G1006fs/49 heterotetramers activation kinetics. The activation curve is significantly left‐shifted by 30 µmol/L NS1643 for both G1006fs/49 homotetramers (C, red trace, cells = 5) and KCNH2‐G1006fs/49 heterotetramers (D, orange trace, cells = 5). In both C and D plots, the green trace is the activation curve for KCNH2 homotetramers. ANOVA test, *P* ≤ 0.05 *G1006fs/49 vs G1006fs/49 NS1643 in C; *P* ≤ 0.05 *KCNH2‐G1006fs/49 vs KCNH2‐G1006fs/49 NS1643 in D

In Figure 7A, G1006Fs/49 homotetramers exhibit a *V*_1/2_ of −6.871 ± 1.812 mV and a slope factor (κ) of 8.072 ± 0.390 in control extracellular solution (red trace) while a *V*_1/2_ of −13.871 ± 1.774 mV and a slope factor (κ) of 8.102 ± 0.210 were recorded after 5‐minute exposure to 30 µmol/L NS1643 (dashed black trace) with a significant left‐shift of about 10 mV. The agonist‐induced shift brought the activation curve of G1006fs/49 homotetramers close to the one previously obtained for the KCNH2 homotetramers (green trace).

In Figure 7B, the K^+^ current recorded for KCNH2‐G1006fs/49 heterotetramers showed a *V*_1/2_ of 10.176 ± 0.49 mV and a slope factor (κ) of 8.5 ± 0.765 in control extracellular solution (orange trace) while a *V*_1/2_ of −10.771 ± 1.872 mV and a slope factor (κ) of 8.674 ± 0.405 were recorded after 10 minutes in 30 µmol/L NS1643 (dashed black trace) resulting in a significant left‐shift of about 20mV of the activation curve, which in turn mostly recapitulates the KCNH2 homotetramer activation kinetics (green trace).

## 4 DISCUSSION

In this work, we functionally characterized a C‐terminal truncating mutant of the KCNH2 channel, G1006fs/49, identified in members of an Italian family affected by LQTS type II.

Specifically, we found that G1006fs/49 mutant (a) is correctly processed in the biosynthetic pathway and localized at the plasma membrane, (b) is able to assemble in functional homo‐ or heterotetrameric channels and (c) exerts a dominant‐negative effect on KCNH2 conferring at the heterotetrameric channel specific biophysical properties, such as a significant delay in the voltage‐sensitive transition to the channel open state, faster inactivation kinetics and a faster recovery from the inactivation. Notably, we found that the altered functionality of the heterotetrameric channel is partially restored by the KCNH2 activator, NS1643.

Several C‐terminal‐truncated KCNH2 mutants have been reported to be functional such as P872fsX5,^12^ R1014X,^13^ G965X, R1014PfsX39 and V1038AfsX21.^14^ These findings are in agreement with a study by Aydar and Palmer, who demonstrated that at least the first 881 amino acids were required for KCNH2 channel functionality.^15^ Interestingly, when expressed in combination with the wild‐type channel subunits the above‐mentioned mutants showed variable capability to act as dominant‐negative, depending on their propensity to form heterotetramers and in turn to modulate either channel trafficking or channel functional properties.

The C‐terminus of KCNH2 contains several regulatory regions such as (a) a cyclic nucleotide binding domain (cNBD) which, interacting with the N‐terminal PAS domain, mediates the slow deactivation kinetics of the channel^16^; (b) an endoplasmic reticulum (ER) retention or RXR signal (residues 1005‐1007), which may regulate intracellular trafficking of the channel^17^; and (c) a conserved coiled‐coil domain (residues 1036‐1074), named TTC, which is essential for subunit assembly/tetramerization.^18^

The mutant G1006fs/49 lacks both the ER retention domain and the coiled‐coil domain. Nevertheless, as shown by confocal analysis, G1006fs/49 mutant shows neither trafficking impairment nor channel inability to assemble as homotetramers or as heterotetramers with KCNH2 channels (Figure 2A,C). Moreover, protein abundance or glycosylation pattern of G1006fs/49 channels indicated no major alterations in intracellular trafficking (Figure 2B). These data are corroborated by the fact that the recorded K^+^ currents of both G1006fs/49 homotetramers and KCNH2‐G1006fs/49 heterotetramers showed a similar amplitude compared to KCNH2 homotetramers (Figure 3A,B,C). Interestingly, both the C‐terminal truncated KCNH2 variants R1014PfsX39 and V1038AfsX21 did not show any disturbance in trafficking and maturation,^14^ in contrast to the mutant R1014X, which had impaired trafficking towards the plasma membrane.^13^ These findings suggest that the novel amino acids introduced by shifts in the reading frame may be still able of masking the ER retention signal in the KCNH2 C‐terminus and of conferring stability to the channel.

The ER retention signal, which is normally masked in the KCNH2 homotetramers, is absent in the G1006fs/49 homotetramers and may be still masked in KCNH2‐G1006fs/49 heterotetramers, by the novel amino acids introduced by shifts in the reading frame. The ability of the KCNH2‐G1006fs/49 heterotetramers to assemble although the lack of the TCC domain in the G1006fs/49 subunits clearly demonstrated, as already reported, that the TCC domain does not play an indispensable role in assembly/tetramerization of KCNH channels.^13^, ^14^, ^19^

What we instead found of novelty in the C‐terminus function mechanisms is that the sequence downstream the position 1006 seems to be important for the voltage sensitivity of the channels. Interestingly, the mutant R1014PfsX39 did not show any alteration in the activation kinetics compared to the WT channel^14^ leading to even narrow the C‐terminal sequence involved in the voltage sensitivity between amino acids 1006‐1014. G1006fs/49 homotetramers and, more strikingly, KCNH2‐G1006fs/49 heterotetramers exhibit a shift of the activation kinetics towards positive voltages and both a faster inactivation kinetics and recovery from the activation. All these biophysical properties are voltage‐sensitive (for review, see 2). Zhao et al proposed an activation model for the KCHN2 channel in which the voltage sensitivity of the gating machinery depends on the interaction of the C‐terminal tail with the N‐terminal PAS domain in the neighbouring subunit.^20^ In this scenario, it is conceivable to hypothesize that our mutation of the channel may induce structural changes in the C‐terminal region in the mutated channel, which can be conveyed, through its interaction with the N‐terminal tail, to the VSD and gating domain in the neighbouring subunits.

Moreover, in the heterotetrameric assembly with KCNH2, the truncated C‐terminal tails of G1006fs/49 subunits exert a dominant‐negative effect over the neighbouring wild‐type channel subunits worsening the biophysical features of the KCNH2‐G1006fs/49 heterotetramers compared to G1006fs/49 homotetramers.

Interestingly, it has been recently demonstrated that the small‐molecule NS1643 interacting with an amino acid within the VSD segment in KCNH2 channels affects the voltage sensor movement on the activation path of the channel.^21^ Accordingly, we and others showed that the treatment with NS1643 was able to sensibly shift the activation curve of KCNH2 towards less positive voltages, thus accelerating the channel opening.^22^, ^23^, ^24^

When we tested the effect of this compound on the biophysical properties of either G1006fs/49 homotetramers or KCNH2‐G1006fs/49 heterotetramers, we found that NS1643 was able to restore the voltage sensitivity activation in both channels, resulting on the left‐shift of their activation curves.

These data not only provide new insights on the role of a specific region of the C‐terminal tail of the KCNH2, but also deeply contribute to improve both the risk stratification and the clinical management of G1006fs/49 carriers.

Prolonged QTc has been identified as major risk factor for cardiac events, and a cut‐off of QTc > 500 ms identifies higher‐risk patients.^25^, ^26^ Moreover, previous observations suggest that the localization of a mutation within the gene or protein may have an impact on clinical presentation in the LQTSs. Interestingly, among hundreds mutations in KCNH2 channel, C‐terminally located mutations have generally been associated with a clinically more benign phenotype compared with those located in the pore region.^27^, ^28^ In this scenario, both the localization of the mutation in the KCNH2 protein (C‐terminus) and the QTc duration in the carriers (QTc < 500 ms in the proband) should have been predictive of a benign disease development. However, the onset of the disease in the mutation carriers resulted severe and typically characterized by cardiac arrest and syncope after either emotional or auditory stresses, respectively.

Of note, in 2007 Moss et al conducted an important study on a large patient cohort with 77 different KCNQ1 mutations leading to LQTS type I. Biophysical properties of the mutations were categorized according to dominant‐negative, haploinsufficiency and reduction in cardiac repolarizing *I*Ks potassium channel current. The obtained results showed that patients with mutations having dominant‐negative functional effect were at increased risk for cardiac events and that these genetic risks were independent of traditional clinical risk factors such as QTc duration, gender and *β*‐blocker therapy.^29^

The functional characterization of the heterotetramers we made in this work, clearly showing the dominant‐negative effect of the G1006fs/49 channel subunits over the wild‐type KCNH2 channel subunits, provided crucial findings about both the pathogenesis of the LQTS2 in the mutant carriers and additional risk information for them beyond the clinical risk factors and the genotype.

We already found that the study of the pathomechanisms underlying other inherited cardiomyopathies is particularly important for the identification of mutation‐specific therapy*.* 30, 31, 32

Moreover, the fact that NS1643 can recapitulate the wild‐type‐like activation kinetics in both G1006fs/49 homo‐ and heterotetramers channels paves a venue for a pharmacological intervention in the patients affected by mutations in the same region of G1006fs/49. Interestingly, in vivo treatment with NS1643 in rabbit models of acquired long QT syndrome due to a dofetilide‐induced KCHN2 inhibition shortened the QT interval in these animals, indicating that this drug therapy is actually able also in vivo to restore the KCHN2 inhibition.^33^

We are aware of the potential limitations of this study. A more meaningful experimental approach would have been to analyse the K^+^ currents described in this paper over cardiomyocytes derived from family carrying the wild‐type and mutated form of KCNH2, respectively. The latter would have taken into account the following key issues: (a) the role of the C‐terminally truncated KCNH2 splicing isoform, Kv11.1a‐USO, in the trafficking of the KCNH2 full‐length isoform towards the plasma membrane in pathophysiological conditions^34^, ^35^ and (b) the contribution of another K^+^ channel recently found to be involved in atrial repolarization such as the voltage‐gated Kv1.1 channel.^36^

However, several factors have made HEK293 cells a popular choice for the study of the KCNH2 biophysics by patch clamp. This cell line allows either studying the biophysical properties of the KCNH2 channel without the interference of other endogenously expressed K^+^ channels or defining the subunits composition in the heterotetrameric expressed channel.

## CONFLICT OF INTEREST

The authors confirm that there are no conflicts of interest.

## AUTHOR CONTRIBUTION

RDZ and AG performed the experiments, analysed the data and contributed to writing the manuscript; CF and MP provided and analysed the clinical data; SM performed the experiments; SF interpreted the clinical data and contributed to writing the manuscript; MS interpreted the experimental data and contributed to writing the manuscript; GP performed the experiments, analysed the data and contributed to writing the manuscript; and MC designed the research study, interpreted the data and wrote the manuscript.

## References

[1] Sanguinetti MC . HERG1 channelopathies. Pflugers Arch. 2010;460:265‐276. 20544339 10.1007/s00424-009-0758-8 PMC2886309

[2] Vandenberg JI , Perry MD , Perrin MJ , Mann SA , Ke Y , Hill AP . hERG K(+) channels: structure, function, and clinical significance. Physiol Rev. 2012;92:1393‐1478. 22988594 10.1152/physrev.00036.2011

[3] Piper DR , Hinz WA , Tallurri CK , Sanguinetti MC , Tristani‐Firouzi M . Regional specificity of human ether‐a'‐go‐go‐related gene channel activation and inactivation gating. J Biol Chem. 2005;280:7206‐7217. 15528201 10.1074/jbc.M411042200

[4] Spector PS , Curran ME , Zou A , Keating MT , Sanguinetti MC . Fast inactivation causes rectification of the IKr channel. J Gen Physiol. 1996;107:611‐619. 8740374 10.1085/jgp.107.5.611 PMC2217012

[5] Smith PL , Baukrowitz T , Yellen G . The inward rectification mechanism of the HERG cardiac potassium channel. Nature. 1996;379:833‐836. 8587608 10.1038/379833a0

[6] Shimizu W , Moss AJ , Wilde AA , et al. Genotype‐phenotype aspects of type 2 long QT syndrome. J Am Coll Cardiol. 2009;54:2052‐2062. 19926013 10.1016/j.jacc.2009.08.028 PMC2808400

[7] Wilde AA , Ackerman MJ . Beta‐blockers in the treatment of congenital long QT syndrome: is one beta‐blocker superior to another? J Am Coll Cardiol. 2014;64:1359‐1361. 25257638 10.1016/j.jacc.2014.06.1192

[8] Nemec J , Ackerman MJ , Tester DJ , Hejlik J , Shen WK . Catecholamine‐provoked microvoltage T wave alternans in genotyped long QT syndrome. Pacing Clin Electrophysiol. 2003;26:1660‐1667. 12877697 10.1046/j.1460-9592.2003.t01-1-00249.x

[9] Sanguinetti MC , Jiang C , Curran ME , Keating MT . A mechanistic link between an inherited and an acquired cardiac arrhythmia: HERG encodes the IKr potassium channel. Cell. 1995;81:299‐307. 7736582 10.1016/0092-8674(95)90340-2

[10] Zhou Z , Gong Q , Ye B , et al. Properties of HERG channels stably expressed in HEK 293 cells studied at physiological temperature. Biophys J. 1998;74:230‐241. 9449325 10.1016/S0006-3495(98)77782-3 PMC1299377

[11] Perissinotti LL , Guo J , De Biase PM , Clancy CE , Duff HJ , Noskov SY . Kinetic model for NS1643 drug activation of WT and L529I variants of Kv11.1 (hERG1) potassium channel. Biophys J. 2015;108:1414‐1424. 25809254 10.1016/j.bpj.2014.12.056 PMC4375712

[12] Paulussen AD , Raes A , Jongbloed RJ , et al. HERG mutation predicts short QT based on channel kinetics but causes long QT by heterotetrameric trafficking deficiency. Cardiovasc Res. 2005;67:467‐475. 15958262 10.1016/j.cardiores.2005.05.017

[13] Puckerin A , Aromolaran KA , Chang DD , et al. hERG 1a LQT2 C‐terminus truncation mutants display hERG 1b‐dependent dominant negative mechanisms. Heart Rhythm. 2016;13:1121‐1130. 26775140 10.1016/j.hrthm.2016.01.012

[14] Choe CU , Schulze‐Bahr E , Neu A , et al. C‐terminal HERG (LQT2) mutations disrupt IKr channel regulation through 14‐3‐3epsilon. Hum Mol Genet. 2006;15:2888‐2902. 16923798 10.1093/hmg/ddl230

[15] Aydar E , Palmer C . Functional characterization of the C‐terminus of the human ether‐à‐go‐go‐related gene K(+) channel (HERG). J Physiol. 2001;534:1‐14. 11432987 10.1111/j.1469-7793.2001.t01-3-00001.x PMC2278693

[16] Gustina AS , Trudeau MC . hERG potassium channel gating is mediated by N‐ and C‐terminal region interactions. J Gen Physiol. 2011;137:315‐325. 21357734 10.1085/jgp.201010582 PMC3047612

[17] Kupershmidt S , Yang T , Chanthaphaychith S , Wang Z , Towbin JA , Roden DM . Defective human Ether‐à‐go‐go‐related gene trafficking linked to an endoplasmic reticulum retention signal in the C terminus. J Biol Chem. 2002;277:27442‐27448. 12021266 10.1074/jbc.M112375200

[18] Jenke M , Sánchez A , Monje F , Stühmer W , Weseloh RM , Pardo LA . C‐terminal domains implicated in the functional surface expression of potassium channels. EMBO J. 2003;22:395‐403. 12554641 10.1093/emboj/cdg035 PMC140720

[19] Gong Q , Keeney DR , Robinson JC , Zhou Z . Defective assembly and trafficking of mutant HERG channels with C‐terminal truncations in long QT syndrome. J Mol Cell Cardiol. 2004;37:1225‐1233. 15572053 10.1016/j.yjmcc.2004.10.002

[20] Zhao Y , Goldschen‐Ohm MP , Morais‐Cabral JH , Chanda B , Robertson GA . The intrinsically liganded cyclic nucleotide‐binding homology domain promotes KCNH channel activation. J Gen Physiol. 2017;149:249‐260. 28122815 10.1085/jgp.201611701 PMC5299623

[21] Guo J , Cheng YM , Lees‐Miller JP , et al. NS1643 interacts around L529 of hERG to alter voltage sensor movement on the path to activation. Biophys J. 2015;108:1400‐1413. 25809253 10.1016/j.bpj.2014.12.055 PMC4375528

[22] Bilet A , Bauer CK . Effects of the small molecule HERG activator NS1643 on Kv11.3 channels. PLoS ONE. 2012;7:e50886. 23226420 10.1371/journal.pone.0050886 PMC3511382

[23] Hansen RS , Diness TG , Christ T , et al. Activation of human ether‐a‐go‐go‐related gene potassium channels by the diphenylurea 1,3‐bis‐(2‐hydroxy‐5‐trifluoromethyl‐phenyl)‐urea (NS1643). Mol Pharmacol. 2006;69:266‐277. 16219910 10.1124/mol.105.015859

[24] Schuster AM , Glassmeier G , Bauer CK . Strong activation of ether‐à‐go‐go‐related gene 1 K+ channel isoforms by NS1643 in human embryonic kidney 293 and Chinese hamster ovary cells. Mol Pharmacol. 2011;80:930‐942. 21856740 10.1124/mol.111.071621

[25] Garson A , Dick M , Fournier A , et al. The long QT syndrome in children. An international study of 287 patients. Circulation. 1993;87:1866‐1872. 8099317 10.1161/01.cir.87.6.1866

[26] Locati ET . QT interval duration remains a major risk factor in long QT syndrome patients. J Am Coll Cardiol. 2006;48:1053‐1055. 16949501 10.1016/j.jacc.2006.06.034

[27] Priori SG , Schwartz PJ , Napolitano C , et al. Risk stratification in the long‐QT syndrome. N Engl J Med. 2003;348:1866‐1874. 12736279 10.1056/NEJMoa022147

[28] Moss AJ , Zareba W , Kaufman ES , et al. Increased risk of arrhythmic events in long‐QT syndrome with mutations in the pore region of the human ether‐a‐go‐go‐related gene potassium channel. Circulation. 2002;105:794‐799. 11854117 10.1161/hc0702.105124

[29] Moss AJ , Shimizu W , Wilde AA , et al. Clinical aspects of type‐1 long‐QT syndrome by location, coding type, and biophysical function of mutations involving the KCNQ1 gene. Circulation. 2007;115:2481‐2489. 17470695 10.1161/CIRCULATIONAHA.106.665406 PMC3332528

[30] Forleo C , Carmosino M , Resta N , et al. Clinical and functional characterization of a novel mutation in lamin a/c gene in a multigenerational family with arrhythmogenic cardiac laminopathy. PLoS ONE. 2015;10:e0121723. 25837155 10.1371/journal.pone.0121723 PMC4383583

[31] Carmosino M , Gerbino A , Schena G , et al. The expression of Lamin A mutant R321X leads to endoplasmic reticulum stress with aberrant Ca(2+) handling. J Cell Mol Med. 2016;20:2194‐2207. 27421120 10.1111/jcmm.12926 PMC5082401

[32] Gerbino A , Bottillo I , Milano S , et al. Functional Characterization of a Novel Truncating Mutation in Lamin A/C Gene in a Family with a Severe Cardiomyopathy with Conduction Defects. Cell Physiol Biochem. 2017;44:1559‐1577. 29197877 10.1159/000485651

[33] Diness TG , Yeh YH , Qi XY , et al. Antiarrhythmic properties of a rapid delayed‐rectifier current activator in rabbit models of acquired long QT syndrome. Cardiovasc Res. 2008;79:61‐69. 18367457 10.1093/cvr/cvn075

[34] Kupershmidt S , Snyders DJ , Raes A , Roden DM . A K^+^ channel splice variant common in human heart lacks a C‐terminal domain required for expression of rapidly activating delayed rectifier current. J Biol Chem. 1998;273:27231‐27235. 9765245 10.1074/jbc.273.42.27231

[35] Mura M , Mehta A , Ramachandra CJ , et al. The KCNH2‐IVS9‐28A/G mutation causes aberrant isoform expression and hERG trafficking defect in cardiomyocytes derived from patients affected by Long QT Syndrome type 2. Int J Cardiol. 2017;240:367‐371. 28433559 10.1016/j.ijcard.2017.04.038

[36] Si M , Trosclair K , Hamilton KA , Glasscock E . Genetic ablation or pharmacological inhibition of Kv1.1 potassium channel subunits impairs atrial repolarization in mice. Am J Physiol Cell Physiol. 2019;316(2):C154‐C161. 30427720 10.1152/ajpcell.00335.2018 PMC6397341

