# Supplementary material for: Functional study of a KCNH2 mutant: Novel insights on the pathogenesis of the LQT2 syndrome
Source: J Cell Mol Med. 2019 Jul 30;23(9):6331–42. doi: 10.1111/jcmm.14521 (PMC6714209; doi:10.1111/jcmm.14521)
Supplement: Supplementary file 1 [file JCMM-23-6331-s001.doc]

**Table 1. Clinical features of family members carrying G1006fs/49**

| **Patient** | **Gender** | **Age*** | **Symptoms** | **Comorbidities** | **ECG findings** | **Echocardiographic parameters** | **Medications** | **ICD implantation** |
| --- | --- | --- | --- | --- | --- | --- | --- | --- |
| I-1 | M | 56 | Asthenia | Diabetes, euthyroid multinodular goiter | Sinus bradycardia,  HR=51 bpm,  PR interval 180 msec, QRS duration 90 msec, QTc interval**†** 498 msec | LVEDD 49 mm  LVEF 62% | Nadolol,  oral hypoglycemic agent |  |
| II-3 | F | 23 | Cardiac arrest due to VF triggered by loud noise during sleep |  | Sinus rhythm,  HR=61 bpm,  PR interval 170 msec, QRS duration 80 msec, QTc interval 484 msec | LVEDD 47 mm  LVEF 60% | Nadolol | Age: 23 years |
| II-5 | M | 11 | Syncope |  | Sinus rhythm,  HR=70 bpm,  PR interval 140 msec, QRS duration 70 msec, QTc interval 572 msec | LVEDD 42 mm  LVEF 60% | Nadolol | Age: 11 years |
| III-3 | F | 6 | Asymptomatic |  | Sinus rhythm,  HR=73 bpm,  PR interval 130 msec, QRS duration 70 msec, QTc interval 463 msec | LVEDD 39 mm  LVEF 72% | Nadolol |  |
| III-4 | M | 4 | Asymptomatic |  | Sinus rhythm,  HR=88 bpm,  PR interval 130 msec, QRS duration 80 msec, QTc interval: 460 msec | LVEDD 34 mm  LVEF 74% | Nadolol |  |

bpm: beats per minute; ECG: electrocardiogram; F = female; HR: Heart Rate; ICD: implantable cardioverter defibrillator; LVEDD = left ventricular end-diastolic diameter; LVEF = left ventricular ejection fraction; M = male; VF: Ventricular Fibrillation.

*At diagnosis/clinical presentation (years)

**†** QTc interval: QT interval corrected for heart rate calculated by Bazett’s formula, where
